# Effective AAV-mediated gene therapy in a mouse model of ethylmalonic encephalopathy

**DOI:** 10.1002/emmm.201201433

**Published:** 2012-08-20

**Authors:** Ivano Di Meo, Alberto Auricchio, Costanza Lamperti, Alberto Burlina, Carlo Viscomi, Massimo Zeviani

**Affiliations:** 1 Unit of Molecular Neurogenetics, The Foundation “Carlo Besta” Institute of Neurology IRCCSMilan, Italy; 2 Telethon Institute of Genetics and Medicine (TIGEM) and Division of Medical Genetics, Department of Pediatrics, “Federico II” UniversityNaples, Italy; 3 Division of Inherited Metabolic Diseases, Department of Pediatrics, University of Padua School of MedicinePadua, Italy

**Keywords:** adeno-associated virus, ethylmalonic encephalopathy, gene therapy, hydrogen sulfide, mitochondrial disease

## Abstract

Ethylmalonic encephalopathy (EE) is an invariably fatal disease, characterized by the accumulation of hydrogen sulfide (H_2_S), a highly toxic compound. *ETHE1*, encoding sulfur dioxygenase (SDO), which takes part in the mitochondrial pathway that converts sulfide into harmless sulfate, is mutated in EE. The main source of H_2_S is the anaerobic bacterial flora of the colon, although in trace amount it is also produced by tissues, where it acts as a ‘gasotransmitter’. Here, we show that AAV2/8-mediated, *ETHE1*-gene transfer to the liver of a genetically, metabolically and clinically faithful EE mouse model resulted in full restoration of SDO activity, correction of plasma thiosulfate, a biomarker reflecting the accumulation of H_2_S, and spectacular clinical improvement. Most of treated animals were alive and well >6–8 months after birth, whereas untreated individuals live 26 ± 7 days. Our results provide proof of concept on the efficacy and safety of AAV2/8-mediated livergene therapy for EE, and alike conditions caused by the accumulation of harmful compounds in body fluids and tissues, which can directly be transferred to the clinic.

## INTRODUCTION

Ethylmalonic encephalopathy (EE; OMIM #602473) is a fatal, early onset, autosomal recessive mitochondrial disease caused by mutations in *ETHE1* (Burlina et al, 1991; Mineri et al, 2008). *ETHE1* encodes a ubiquitous mitochondrial sulfur dioxygenase (SDO) (Tiranti et al, 2009) involved in the detoxification of H_2_S (Hildebrandt & Grieshaber, 2008), which is produced in tissues by the catabolism of sulfurated amino acids (Kabil & Banerjee, 2010) and, in the large intestine, by anaerobic bacteria (Flannigan et al, 2011) (Supporting Information Fig S1A). In trace amounts, H_2_S, an elusive, highly volatile gas, is involved in the regulation of the vessel tone and, possibly, in neurotransmission (Gadalla & Snyder, 2010). However, at higher concentrations it acts as a pleiotropic, powerful poison of several enzymes, such as cytochrome c oxidase (COX) (Di Meo et al, 2011) and short chain acyl-CoA dehydrogenase (SCAD) (Tiranti et al, 2009), and directly damages the vascular endothelium (Giordano et al, 2012). These deleterious effects explain the main signs and symptoms of human EE, namely, rapidly progressive neurological failure due to the accumulation of multiple necrotic and haemorrhagic brain lesions (Yang et al, 2004), chronic haemorrhagic diarrhoea, vascular petechial purpura and orthostatic acrocyanosis. Biochemically, EE is characterized by high plasmatic and urinary levels of thiosulfate, a stable, easily measurable compound that directly reflects accumulation of H_2_S (Furne et al, 2001), and ethylmalonic acid (EMA), the carboxylated derivative of butyrate, which reflects the block of oxidative catabolism of butyryl-CoA (Corydon et al, 1996), by H_2_S-mediated inhibition of SCAD. In addition, generalized, H_2_S-mediated COX deficiency occurs in muscle, brain and colonic mucosa (Di Meo et al, 2011). These clinical and biochemical features are faithfully recapitulated in a recombinant *Ethe1*^*−/−*^ mouse model (Tiranti et al, 2009).

Effective therapy for EE must aim at either reducing H_2_S production, increasing its clearance and detoxification, or both. This rationale underpinned a partially successful, however palliative, treatment, applied to both EE patients and *Ethe1*^*−/−*^ mice, based on administration of either N-acetylcysteine (NAC), a precursor of H_2_S-buffering glutathione, or metronidazole, a bactericidal agent specific against anaerobic bacteria, or both (Viscomi et al, 2010). Here, we show the results of a strategy based on liver-restricted adeno-associated virus (AAV)-mediated gene-replacement.

## RESULTS

Using an AAV2/8 vector expressing GFP under the liver-specific thyroxine-binding globulin (TBG) promoter, we first established that the most effective route was intra-cardiac (i.c.) injections in avertin-anesthetized 21 day-old mice. Tail-vein injection was unfeasible due to the small size of the animals, and the intra-peritoneal route was ineffective, likely because of the imperviousness of the peritoneum to viral particles.

Next, we generated an AAV2/8 vector expressing human HA-tagged wild-type (wt) *ETHE1* (*h.ETHE1*^*HA*^) cDNA (Tessitore et al, 2008) (Supporting Information Fig S1B). We then i.c. injected 4 × 10^12^ AAV2/8-TBG-*h.ETHE1*^*HA*^ viral genomes (vg)/kg of body weight in four, 21-day old (P21) *Ethe1*^*−/−*^ mice that had been given 1% NAC in drinking water since P18 to prevent rapid catastrophic downhill development of the clinical conditions occurring in naïve untreated *Ethe1*^*−/−*^ individuals (Viscomi et al, 2010). Mice were euthanized at P35. The *h.ETHE1*^*HA*^ cDNA and protein, as well as SDO activity, were detected exclusively in the liver (Supporting Information Fig S2A–B). Both protein amount and enzymatic activity reached approximately 60% of those in wt littermates. However, this partial biochemical correction was ineffective in significantly prolonging the lifespan of a second group of four (AAV + NAC)-treated *Ethe1*^*−/−*^ mice, compared to that of *Ethe1*^*−/−*^ littermates exposed only to NAC (Supporting Information Fig S2C). Accordingly, plasma thiosulfate remained very high in both groups (Supporting Information Fig S2D).

**Figure 1:**
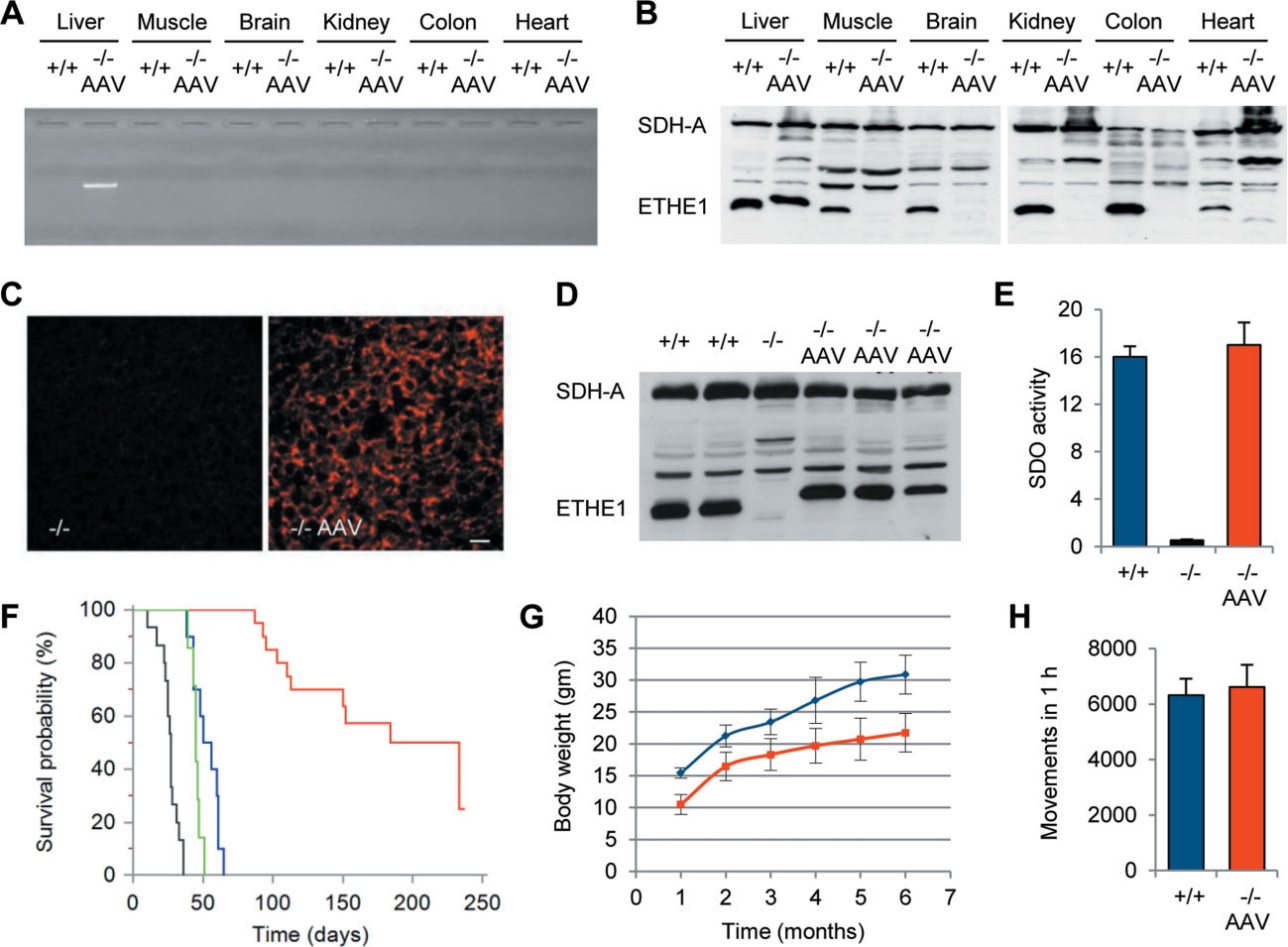
Molecular and clinical characterization of AAV2/8-TBG-*h.Ethe1^HA^*-treated mice A single i.c. injection of 4 × 10^13^ vg/kg was performed in each P21 animal. PCR analysis of AAV2/8 tissue distribution.Western-blot analysis of tissue homogenates using an anti-(α)-ETHE1 antibody: the h.ETHE1^HA^ protein is detected only in the liver of AAV-treated animals as a band slightly slower than that corresponding to the endogenous mouse Ethe1 protein present in all wt (+/+) tissues. SDH-A, the 70 kDa subunit of succinate dehydrogenase is used as a protein-loading standard.Immunofluorescence of liver using an α-HA antibody. The h.ETHE1^HA^ protein is diffusely distributed in the liver of AAV-treated animals (−/− AAV, right panel), whereas it is absent in *naïve* −/− animals (left panel). Scale bar: 50 µm.Western-blot analysis of h.ETHE1^HA^ protein in liver: the amount detected in three AAV-treated mice (−/− AAV) is comparable to that of wt littermates (+/+), whereas the protein is absent in a sample from a *naïve* −/− animal.SDO activity in liver expressed in nmol O_2_/min/mg: the activity of AAV-treated *Ethe1*^*−/−*^ samples (−/− AAV; *red bar*; *n* = 3) is comparable to that of wt samples (+/+; *blue bar*; *n* = 2). Note that SDO activity is virtually absent in untreated *Ethe1*^*−/−*^ samples (−/−; *black bar*; *n* = 3).Kaplan–Meier survival probability graph. Significance was assessed by log-rank test. *Grey*: untreated *Ethe1*^*−/−*^ mice (*n* = 15); *green*: *Ethe1*^*−/−*^ mice treated with NAC for 10 days (*n* = 7); *blue*: *Ethe1*^*−/−*^ treated with NAC *ad libitum* (*n* = 10); *red*: AAV-treated *Ethe1*^*−/−*^ mice (*n* = 20).Variation of body weights over time: for each point the difference between AAV-treated *Ethe1*^*−/−*^ mice (*red*) *versus* wt littermates (*blue*) was significant (*p* < 0.001).Activity cage test: no significant difference in spontaneous locomotor activity was measured between −/− AAV-treated mice (*n* = 11) *versus* wt (+/+) littermates at 3–5 months of age.

In a second pilot experiment, we administered a ten-fold higher titre (4 × 10^13^ vg/kg) of AAV2/8-TBG-*h.ETHE1*^*HA*^ to three NAC-treated P21 *Ethe1*^*−/−*^ mice. Again, *h.ETHE1*^*HA*^ cDNA and protein was present only in, and evenly distributed throughout, the liver (Fig 1A–C), but expression, amount and activity were now comparable to those of wt littermates (Fig 1E–D). We then expanded the 4 × 10^13^ vg/kg treatment to ten additional, NAC-treated *Ethe1*^*−/−*^ mice at P21. This dosage resulted in marked amelioration of the clinical conditions, reflected by highly significant prolongation of the lifespan. Five animals survived up to 4–6 months and were sacrificed for autoptic examination when still alive and well. The remaining five died during the observation time at an age ranging between 3 and 4 months. The median survival of ‘NAC-only’ treated *Ethe1*^*−/−*^ mice was less than 2 months (log-rank test *p* < 0.0001) (Supporting Information Fig S3A).

In a second trial, ten *Ethe1*^*−/−*^ mice were treated with the same protocol and dosage, but NAC administration was suspended at P28. Survival was monitored up to 8 months. Again, of the 6 individuals that offset 7 months of age, five were alive and well at the time of sacrifice, whereas one died at >7 months; one individual died at 6 months; two at 5 months; one at 3. The median survival of naive untreated *Ethe1*^*−/−*^ mice (with no NAC) was <1 month (log-rank test *p* < 0.0001) (Supporting Information Fig S3B).

The survival probability between the two AAV-treated groups did not differ significantly; a cumulative Kaplan–Meier distribution of survival probability is shown in Fig 1F. Lifespan prolongation was accompanied by increased body-weight over time, which was similar to, albeit less than, wt littermates (Fig 1G). In sharp contrast with untreated *Ethe1*^*−/−*^ mice, 3–5 month-old AAV-treated animals (*n* = 11) showed a striking increase of spontaneous motor activity, comparable to that of wt littermates (*n* = 9) (Fig 1H).

**Figure 2:**
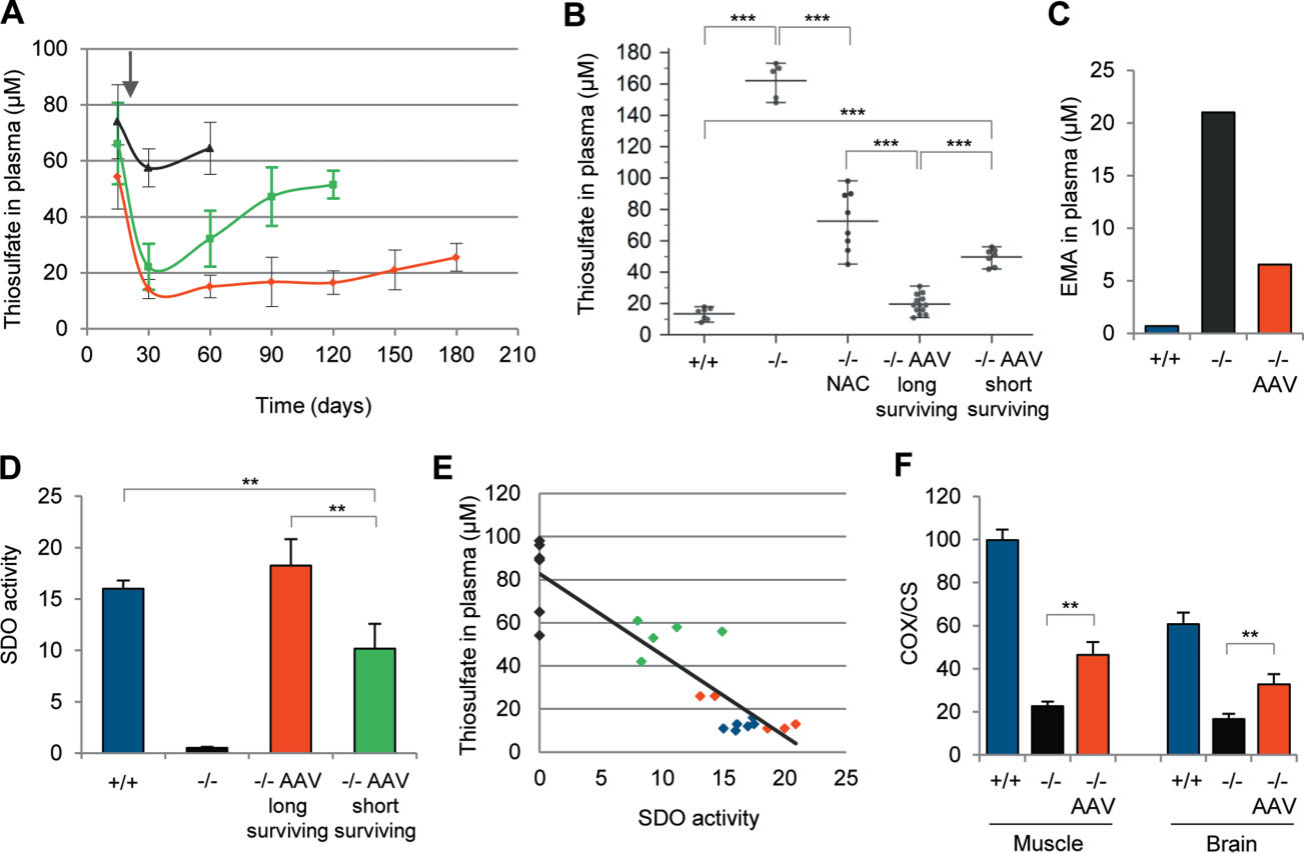
Biochemical analysis Plasma thiosulfate measured at different time-points in shorter- (*green*) and longer- (*red*) surviving *Ethe1*^*−/−*^ individuals treated with 4 × 10^13^ vg/kg AAV2/8-TBG-*h.ETHE1*^*HA*^, and in NAC-only treated *Ethe1*^*−/−*^ mice (*gray*). The arrow indicates the time of the AAV injection (P21).Thiosulfate concentration at the last time-point before death. In longer-surviving AAV-treated *Ethe1*^*−/−*^ animals, the thiosulfate concentration is comparable to that of the wt ones, whereas in shorter-surviving AAV-treated animals it is comparable to that of the ‘NAC-only’ treated ones. ***: unpaired Student's *t*-test *p* < 0.0001.Plasma EMA in pooled samples.SDO activities expressed as nmol O_2_/min/mg in wt controls (+/+; *blue bar*; *n* = 3); untreated *Ethe1*^*−/−*^ animals (*black bar*; *n* = 3); longer-surviving AAV-treated animals (*red bar*; *n* = 5) and shorter-surviving AAV-treated animals (*green bar*; *n* = 5). **: Student's *t*-test *p* = 0.008.Correlation analysis of plasma thiosulfate *versus* liver SDO activity. *Black diamonds*: ‘NAC-only’ treated *Ethe1*^*−/−*^; *green*: AAV-treated, shorter-surviving *Ethe1*^*−/−*^; *red*: AAV-treated, longer-surviving *Ethe1*^*−/−*^; *blue*: *Ethe1*^*+/+*^. *χ*^2^-test correlation *R*^2^ = 0.82 (*p* = 0.001).COX/CS activity in muscle and brain of wt controls (+/+; *blue bar*; *n* = 3); untreated *Ethe1*^*−/−*^ animals (*black bar*; *n* = 3); AAV-treated animals (*red bar*; *n* = 5) **: Student's *t*-test *p* = 0.004.

The levels of plasma thiosulfate reflected the lifespan, and in fact predicted the outcome, of AAV-treated individuals (Fig 2A), since those that displayed consistent correction to nearly normal levels (19.0 ± 6.0 µM; wt control mean: 14.0 ± 4.0 µM; NS) lived much longer than those showing a progressive increase (51.0 ± 4.6 µM; *p* < 0.0001) (Fig 2B). Plasma EMA in the AAV-treated group was much lower than that in the untreated group (Fig 2C). SDO activity in liver mitochondria of AAV-treated mice was virtually normal in longer-surviving (18.2 ± 2.6 nmol O_2_/min/mg; normal values: 16.0 ± 0.8; NS), but lower-than-normal in shorter-surviving, individuals (10.2 ± 2.4; *p* = 0.001) (Fig 2D). Thiosulfate levels in plasma correlated with liver SDO activity (*R*^2^ = 0.82, *p* < 0.0001) (Fig 2E), and AAV DNA copy number in hepatocytes was significantly higher in longer- than in shorter-surviving groups (24.6 ± 2.5 AAV DNA copies/diploid genome vs. 10.4 ± 2.0; *p* = 0.007). The amount of thiosulfate was much lower in tissues than in plasma, and for some tissues, such as brain, it was below the detectability threshold (Tiranti et al, 2009). Nevertheless, we found a significant reduction of thiosulfate levels in muscle homogenates from AAV-treated *Ethe1*^*−/−*^ animals compared to untreated *Ethe1*^*−/−*^ individuals (Supporting Information Fig S4), which suggests that the clearance of circulating H_2_S is effective in reducing its concentration in critical, extrahepatic tissues.

Biochemical assays for COX activity normalized to that of citrate synthase (CS) showed significant recovery in both skeletal muscle and brain of treated *versus* untreated animals (Fig 2F and Supporting Information Table S1).

**Figure 3:**
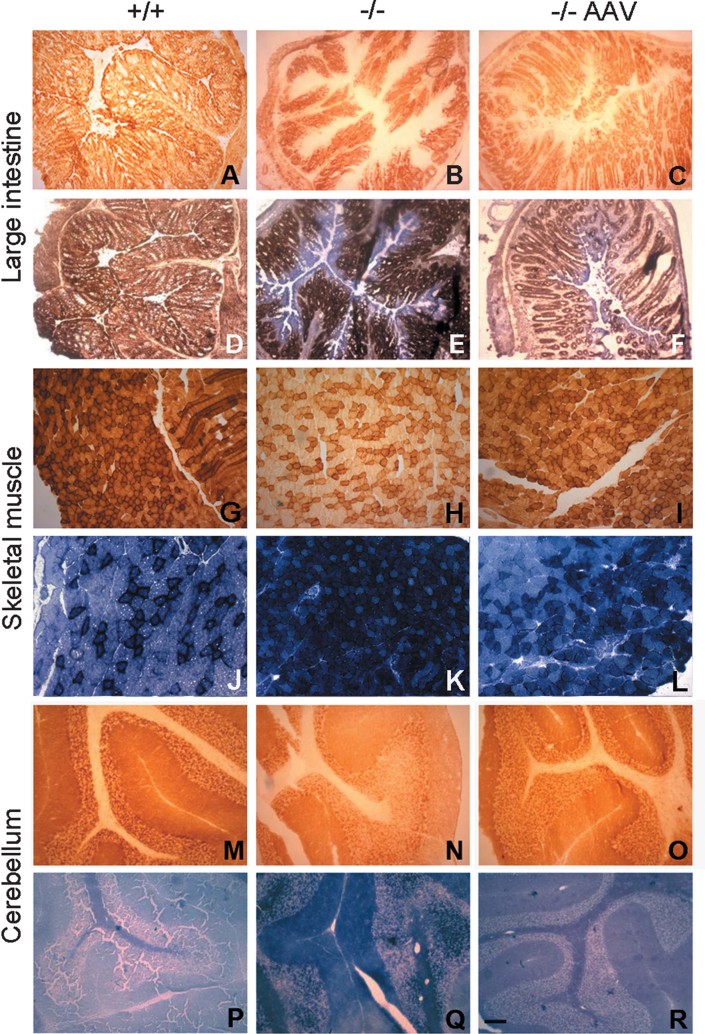
Histochemical analysis Frozen sections from a 6 month-old Ethe1^+/+^ (+/+), a 1 month-old Ethe1^−/−^ (−/−), and a 6 month-old AAV-treated Ethe1^−/−^ (−/− AAV) mouse. **A–C.** COX reaction.**D–F.** Combined COX + SDH reactions in the large intestine.**G–I.** COX reaction.**J–L.** SDH reaction based on the conversion of tetrazolium into blue formazan.**M–O.** COX reaction.**P–R.** SDH reaction based on the conversion of tetrazolium into blue formazan. Scale bar: 50 mm. See text for details.

Histochemical reaction to COX and SDH was performed in skeletal muscle, brain (cerebellum), and large intestine of *Ethe1*^*+/+*^, *Ethe1*^*−/−*^ and AAV-treated *Ethe1*^*−/−*^ mice (*n* = 3 for each group). Whilst naive *Ethe1*^*−/−*^ mice displayed marked COX deficiency due to H_2_S poisoning (Di Meo et al, 2011) in the luminal surface of the colonic mucosa, muscle fibres and brain cells, the AAV treatment was associated with clearly visible, albeit partial, correction to nearly normal levels (Fig 3). Likewise, markedly hyperintense ‘SDH-like’ blue staining that was evident in naive *Ethe1*^*−/−*^ mice returned to nearly normal levels in tissues from AAV-treated *Ethe1*^*−/−*^ mice (Fig 3). We have previously shown that hyper intense blue staining in *Ethe1*^*−/−*^ tissues is a ‘spurious’ reaction caused by direct H_2_S-mediated reduction of colourless tetrazolium, the histochemical SDH substrate, into blue formazan (Di Meo et al, 2011). Thus, the lesser intense blue reaction in AAV-treated tissues reflects lower H_2_S concentration *in situ*.

**The paper explained**

PROBLEM:

Ethylmalonic encephalopathy (EE) is an invariably fatal disease characterized by the accumulation of hydrogen sulfite (H_2_S), a highly toxic compound. The main source of H_2_S is the anaerobic bacterial flora of the colon, although it is also produced by tissues in trace amounts, where it acts as a “gasotransmitter”. EE is caused by mutations in *ETHE1*, a gene encoding a mitochondrial sulfur dioxygenase (SDO), which takes part in the pathway that converts sulfite into harmless sulfate. We have previously proposed a pharmacological therapy based on the off-label use of approved drugs (Metronidazole and NAC) to lower the production and promote the intra-cellular detoxification of H_2_S. Although beneficial, this palliative therapy is far from being curative.

RESULTS:

In order to develop a more effective, etiological therapy for EE, we have used AAV-mediated gene targeting to express the human ETHE1 protein *in vivo*. AAV2/8-mediated *ETHE1*-gene transfer to the liver of a genetically, metabolically and clinically faithful EE mouse model resulted in full restoration of *in vitro* SDO activity, correction of plasma thiosulfate, a biomarker reflecting the accumulation of H_2_S, and efficient clinical improvement. Most of the treated animals were alive and well >6–8 months after birth, whereas untreated individuals lived 26 ± 7 days.

IMPACT:

Our results provide proof of concept on the efficacy and safety of AAV2/8-mediated liver gene therapy for EE and alike conditions caused by the accumulation of harmful compounds in body fluids and tissues, which can directly be transferred to the clinic.

## DISCUSSION

The key-pathomechanism of EE is the accumulation of H_2_S in the bloodstream and tissues up to toxic levels, which eventually inhibits crucial enzymes such as COX and SCAD and directly damages the endothelial lining. We reasoned that the clearance of circulating H_2_S, by expressing the missing *ETHE1* gene in a filtering organ such as the liver, could decrease the levels of H_2_S, thus acting as a detoxifying means for effective disease treatment. To test this hypothesis, we used an AAV2/8-TBG vector to express *h.ETHE1*^*HA*^ in the liver of *Ethe1*^*−/−*^ mice. After optimization of route and titer, we then treated a total of 20 animals that all showed marked amelioration of the phenotype and spectacular prolongation of the lifespan. This remarkable clinical result was associated with partial or complete correction of the main metabolic and biochemical indexes of disease, including EMA and thiosulfate levels in plasma as well as COX activity in tissues. Interestingly, the levels of thiosulfate, a stable biomarker mirroring the amount of labile H_2_S, were much higher in plasma of untreated *Ethe1*^*−/−*^ animals than in muscle homogenates, suggesting that circulating H_2_S can have more prominent and widespread damaging effects than H_2_S produced endogenously in individual tissues. This is concordant with the observation that conditional brain- or muscle-specific *Ethe1*^*−/−*^ mice display hardly any tissue damage or clinical effect (Di Meo et al, 2011). We conclude that liver-specific AAV-based treatment can correct, at least partially, H_2_S-mediated inhibition of SCAD and COX in brain, muscle and colonic mucosa, by just lowering H_2_S concentration in the bloodstream, as reflected by normalization (or marked decrease) of circulating thiosulfate. We propose that the restoration of H_2_S detoxifying competency by the hepatic filter can prevent the most ominous pathological feature in human EE, *i.e*. the direct damage of endothelia in critical organs (Giordano et al, 2012) caused by circulating H_2_S (Yang et al, 2004). Unfortunately, this hypothesis is difficult to test in *Ethe1*^*−/−*^ mice, whose vascular lesions, possibly because of their very short lifespan, are much less prominent than those of EE patients (Giordano et al, 2012).

Long-term therapeutic levels of coagulation Factor IX have recently been achieved in hemophilia B patients treated with a single intravascular administration of the recombinant AAV2/8 vector (Nathwani et al, 2011). Likewise, our results open realistic perspectives to the treatment of human EE, providing proof-of-principle, evidence-based demonstration that AAV-driven *ETHE1* gene therapy is an effective and feasible approach, directly translatable to clinical practice.

In a broader perspective, the same strategy can be applied to other conditions such as mitochondrial neuro-gastro-intestinal encephalomyopathy (MNGIE) (Nishino et al, 1999) caused by the accumulation of thymidine up to mutagenic levels for mtDNA.

## MATERIALS AND METHODS

### Construction of AAV2/8 vectors

AAV2/8-TBG-*h.ETHE1*^*HA*^ and AAV2/8-TBG-*eGFP* vectors were produced by the AAV Vector Core of the Telethon Institute of Genetics and Medicine (TIGEM, Naples, Italy) by triple transfection of 293 cells and purified by CsCl gradients (Xiao et al, 1999). Physical titers of the viral preparations (genome copies/mL) were determined by real-time PCR (Gao et al, 2000) (Applied Biosystems, Foster City, CA, USA) and dot-blot analysis.

### Genomic DNA extraction, PCR and quantitative PCR

Total DNA was extracted from frozen tissues (Viscomi et al, 2009). AAV-derived DNA was detected by standard PCR amplification using primer pairs specific to the *h.ETHE1* gene. SYBR-GREEN based real-time quantitative PCR was carried out as previously described (Di Meo et al, 2011) using primers specific to the *h.ETHE1* gene; the RNAseP gene was used as a reference. Oligonucleotide sequences are available on request.

### Immunoblotting

Western-blot analysis was performed on mouse tissue homogenates with an α-ETHE1 (Tiranti et al, 2004) and an α-succinate dehydrogenase-A (α-SDHA; 0.1 µg/mL) using the ECL chemiluminescence kit (Amersham) (Di Meo et al, 2011).

### Immunofluorescence, histochemical and biochemical analyses

Immunofluorescence analysis was carried out on cryostat sections fixed with methanol using an α-HA polyclonal primary antibody (Abcam). Standard histochemical reactions for COX and SDH were performed on cryostat sections (Sciacco & Bonilla, 1996). SDO activity in liver and thiosulfate in plasma and tissues were measured as described (Hildebrandt & Grieshaber, 2008). Plasma EMA was assessed by GC/MS (Wilcken et al, 2003). Spectrophotometric analysis was carried out as described (Di Meo et al, 2011).

### Statistical analysis

For comparison between groups we used two-tailed, unpaired Student's *t*-test. Correlation significance was determined by *χ*^2^ test. Survival probability analysis was calculated using Kaplan–Meier estimate and log-rank test.

### Experimental ethics policy

Animal studies were approved by the Ethics Committee of the Foundation ‘Carlo Besta’ Neurological Institute, in accordance with the guidelines of the Italian Ministry of Health. The use and care of animals followed the Italian Law D.L. 116/1992 and the EU directive 86/609/CEE. The mice were kept on a C57Bl6/129Sv mixed background, and wt littermates were used as controls. Standard food and water were given *ad libitum*.

## For more information

TELETHON-Italy Foundation official website: http://www.telethon.it

Association Francaise contre les Myopathies (AFM) official website: http://www.afm-telethon.fr

TIGEM (Telethon Institute of Genetic Medicine) official website: http://www.tigem.it

Pierfranco and Luisa Mariani Center for the Study of Paediatric Mitochondrial Disease: http://www.mitopedia.org

Mitocon-Italian Association of Mitochondrial Disease Patients: http://www.mitocon.it

OMIM Online Mendelian Inheritance in Man: http://www.ncbi.nlm.nih.gov/omim/

National Center for Biotechnology Information: http://www.ncbi.nlm.nih.gov

The Foundation Carlo Besta Institute of Neurology – IRCCS official website: http://www.istituto-besta.it

Clinical Trials on AAV-mediated gene replacement can be found at: http://www.clinicaltrials.gov

## Supplementary material

Detailed facts of importance to specialist readers are published as ”Supporting Information”. Such documents are peer-reviewed, but not copy-edited or typeset. They are made available as submitted by the authors.

## References

[b1] Burlina A, Zacchello F, Dionisi-Vici C, Bertini E, Sabetta G, Bennet MJ, Hale DE, Schmidt-Sommerfeld E, Rinaldo P (1991). New clinical phenotype of branched-chain acyl-CoA oxidation defect. Lancet.

[b2] Corydon MJ, Gregersen N, Lehnert W, Ribes A, Rinaldo P, Kmoch S, Christensen E, Kristensen TJ, Andresen BS, Bross P (1996). Ethylmalonic aciduria is associated with an amino acid variant of short chain acyl-coenzyme A dehydrogenase. Pediatr Res.

[b3] Di Meo I, Fagiolari G, Prelle A, Viscomi C, Zeviani M, Tiranti V (2011). Chronic exposure to sulfide causes accelerated degradation of cytochrome c oxidase in ethylmalonic encephalopathy. Antioxid Redox Signal.

[b4] Flannigan KL, McCoy KD, Wallace JL (2011). Eukaryotic and prokaryotic contributions to colonic hydrogen sulfide synthesis. Am J Physiol Gastrointest Liver Physiol.

[b5] Furne J, Springfield J, Koenig T, DeMaster E, Levitt MD (2001). Oxidation of hydrogen sulfide and methanethiol to thiosulfate by rat tissues: a specialized function of the colonic mucosa. Biochem Pharmacol.

[b6] Gadalla MM, Snyder SH (2010). Hydrogen sulfide as a gasotransmitter. J Neurochem.

[b7] Gao G, Qu G, Burnham MS, Huang J, Chirmule N, Joshi B, Yu QC, Marsh JA, Conceicao CM, Wilson JM (2000). Purification of recombinant adeno-associated virus vectors by column chromatography and its performance in vivo. Hum Gene Ther.

[b8] Giordano C, Viscomi C, Orlandi M, Papoff P, Spalice A, Burlina A, Di Meo I, Tiranti V, Leuzzi V, d'Amati G (2012). Morphologic evidence of diffuse vascular damage in human and in the experimental model of ethylmalonic encephalopathy. J Inherit Metab Dis.

[b9] Hildebrandt TM, Grieshaber MK (2008). Three enzymatic activities catalyze the oxidation of sulfide to thiosulfate in mammalian and invertebrate mitochondria. FEBS J.

[b10] Kabil O, Banerjee R (2010). Redox biochemistry of hydrogen sulfide. J Biol Chem.

[b11] Mineri R, Rimoldi M, Burlina AB, Koskull S, Perletti C, Heese B, von Döbeln U, Mereghetti P, Di Meo I, Invernizzi F (2008). Identification of new mutations in the ETHE1 gene in a cohort of 14 patients presenting with ethylmalonic encephalopathy. J Med Genet.

[b12] Nathwani AC, Tuddenham EG, Rangarajan S, Rosales C, McIntosh J, Linch DC, Chowdary P, Riddell A, Pie AJ, Harrington C (2011). Adenovirus-associated virus vector-mediated gene transfer in hemophilia B. N Engl J Med.

[b13] Nishino I, Spinazzola A, Hirano M (1999). Thymidine phosphorylase gene mutations in MNGIE, a human mitochondrial disorder. Science.

[b14] Sciacco M, Bonilla E (1996). Cytochemistry and immunocytochemistry of mitochondria in tissue sections. Methods Enzymol.

[b15] Tessitore A, Faella A, O'Malley T, Cotugno G, Doria M, Kunieda T, Matarese G, Haskins M, Auricchio A (2008). Biochemical, pathological, and skeletal improvement of mucopolysaccharidosis VI after gene transfer to liver but not to muscle. Mol Ther.

[b16] Tiranti V, D'Adamo P, Briem E, Ferrari G, Mineri R, Lamantea E, Mandel H, Balestri P, Garcia-Silva MT, Vollmer B (2004). Ethylmalonic encephalopathy is caused by mutations in ETHE1, a gene encoding a mitochondrial matrix protein. Am J Hum Genet.

[b17] Tiranti V, Viscomi C, Hildebrandt T, Di Meo I, Mineri R, Tiveron C, Levitt MD, Prelle A, Fagiolari G, Rimoldi M (2009). Loss of ETHE1, a mitochondrial dioxygenase, causes fatal sulfide toxicity in ethylmalonic encephalopathy. Nat Med.

[b18] Viscomi C, Burlina AB, Dweikat I, Savoiardo M, Lamperti C, Hildebrandt T, Tiranti V, Zeviani M (2010). Combined treatment with oral metronidazole and N-acetylcysteine is effective in ethylmalonic encephalopathy. Nat Med.

[b19] Viscomi C, Spinazzola A, Maggioni M, Fernandez-Vizarra E, Massa V, Pagano C, Vettor R, Mora M, Zeviani M (2009). Early-onset liver mtDNA depletion and late-onset proteinuric nephropathy in Mpv17 knockout mice. Hum Mol Genet.

[b20] Wilcken B, Wiley V, Hammond J, Carpenter K (2003). Screening newborns for inborn errors of metabolism by tandem mass spectrometry. N Engl J Med.

[b21] Xiao W, Chirmule N, Berta SC, McCullough B, Gao G, Wilson JM (1999). Gene therapy vectors based on adeno-associated virus type 1. J Virol.

[b22] Yang G, Sun X, Wang R (2004). Hydrogen sulfide-induced apoptosis of human aorta smooth muscle cells via the activation of mitogen-activated protein kinases and caspase-3. FASEB J.

