# Supplementary material for: Effective AAV-mediated gene therapy in a mouse model of ethylmalonic encephalopathy
Source: EMBO Mol Med. 2012 Aug 20;4(9):1008–14. doi: 10.1002/emmm.201201433 (PMC3491831; doi:10.1002/emmm.201201433)
Supplement: Supplementary file 1 [file emmm0004-1008-SD1.pdf]

EMBO
Molecular Medicine

Manuscript EMM-2012-01433

Effective AAV-mediated gene therapy in a mouse model of Ethylmalonic Encephalopathy

Ivano Di Meo, Alberto Auricchio, Costanza Lamperti, Alberto Burlina, Carlo Viscomi, and Massimo Zeviani

Corresponding author: Massimo Zeviani, The Foundation "Carlo Besta" Institute of Neurology IRCCS

Review timeline:

Submission date:	05 April 2012
Editorial Decision:	15 May 2012
Revision received:	08 June 2012
Editorial Decision:	29 June 2012
Revision received:	02 July 2012
Accepted:	03 July 2012

Transaction Report:

(Note: With the exception of the correction of typographical or spelling errors that could be a source of ambiguity, letters and reports are not edited. The original formatting of letters and referee reports may not be reflected in this compilation.)

1st Editorial Decision 15 May 2012

Thank you for the submission of your manuscript "Remarkably effective AAV-mediated gene therapy in a mouse model of Ethylmalonic Encephalopathy". We have now heard back from the three referees whom we asked to evaluate your manuscript. You will see that they find the topic of your manuscript potentially interesting but they feel that the data need to be strengthened, which should be addressed in a major revision.

In particular, reviewer #1 highlights that measurements of respiratory chain activity as well as H2S in tissues after rescue should be performed. In addition, both reviewers #2 and #3 note the absence of important controls that should be included.

Given the balance of the evaluations, we would be willing to consider a revised manuscript with the understanding that the reviewers' concerns must be convincingly addressed within the time constraints outlined below.

Revised manuscripts should be submitted within three months of a request for revision. They will otherwise be treated as new submissions, unless arranged otherwise with the editor.

I look forward to seeing a revised form of your manuscript as soon as possible.

Yours sincerely,

Editor
EMBO Molecular Medicine

***** Reviewer's comments *****

Referee #1:

The authors present a convincing case that a mouse model of ethylmalonic aciduria can be treated with a recombinant human viral vector.

Figure 1D. We need to see SDO levels in untreated mice, to be convinced that the mechanism is due to the restoration of SDO activity.
Ibid in Fig 2D
How do the authors explain the multi-tissue rescue implied by Figure 3, given the highly tissue specific expression of the human protein shown in Figure 1A? Presumably ALL of the non-hepatic effects are due to the removal of H2S, which creates the impression of improved OXPHOS? To be convincing, we really need to see formal measurement of the respiratory chain activity in these tissues, and parallel measures of H2S in these tissues. At present we are left with uncertainty on this issue.
Have the authors tried allogenic bone marrow transplantation in their mice?

The title should not include "remarkably", which is a subjective term.

Referee #2:

This paper by Di Meo et al describes a new strategy of treatment of ethylmalonic encephalopathy, a so far always fatal disease in humans, in a mouse model of the disease where the Ethe1 gene has been invalidated, by using AAV2/8 viruses to transduce the liver cells of the mice with the wild type human ETHE1 gene. Authors provide convincing evidence that the treatment of Ethe1-/- KO mice with AAV2/8-ETHE1 viruses allows Ethe1-/- mice to survive much longer than untreated mice, can restore their SDO enzymatic activity in liver to normal levels, as well as their blood level of thiosulfate, which in the disease directly correlates with the accumulation of toxic H2S due to deficiency in SDO activity.
The paper is clear and well written, and provides very interesting perspectives of clinical trials in patients by the use of similar AAV vectors. The referee feels that this manuscript will become suitable for publication in EMBO Molecular Medicine provided that authors answer the following recommandations:

- Authors used the strategy to target liver as the main filtering organ after blood circulation from intesine, one of the major productor of H2S. However, as said in the manuscript, H2S is also produced (at much lower amounts) to be used in many tissues, including CNS, as "gazotransmitter". Do authors know how, if yes, ETHE1 is expressed in the brain, and whether the invalidation of ETHE1 expression in the brain of patients may be responsible of the neurological symptoms independently of the liver defect? This concept is emerging now for numerous inborn errors of metabolism, and may be very important to consider for the long term outcome of patients. In particular, would excess production of H2S in CNS be evacuated to the bloodstream through blood-brain barrier, or would this excess H2S be trapped in the brain and be harmful here directly? In case of an endogenous ETHE1 expression in CNS, correction of the liver enzyme by AAV2/8 may appear insufficient to treat patients on the long term range. Authors may comment on this in their discussion.

- How are the AAV2/8 viruses purified in terms of full viruses (including genome) and empty particles? Did authors analyse the potential effects of control (i.e. non-transducing ETHE1) AAV2/8 viruses in their mouse model? AAV2/8 per se may have toxic effects in various tissues, and this very important control seems to lack in the manuscript.

- Kaplan-Meier survival curves in supplementary figures 1 and 2 should be better explained, in particular what represent the different colored curves.

- Supplementary figure 1B: please add that this is (probably) a liver extract.

Referee #3:

EE is an autosomal recessive fatal disorder due to mutations in ETHE1, which encodes sulfur dioxygenase, a mitochondrially-targeted protein involved in H2S detoxification. Mice lacking Ethe1 die within a few weeks. The authors report here of a dramatic increase in lifespan and in various aspects of Ethe1-related biochemistry and morphology in mice treated with a high titer of AAV expressing WT-Ethe1 under a liver-specific promoter.

This is a really nice paper demonstrating the potential and power of targeted gene therapy to treat, and perhaps even cure, an otherwise fatal monogenic disorder. The work has been done well and is convincing. I have only a few comments, mainly for the benefit of the reader.

Although reported elsewhere, it would be helpful to show, probably in Fig. 1, the pathway of H2S detoxification and where ETHE1/SDO fits into this scheme. Similarly, it would be informative to show the AAV construct in general outline (e.g. vector, promoter, gene, any relevant regulatory elements, location of pCR primers used to confirm DNA/mRNA).

Please note in the introduction in what tissues SDO is normally synthesised.

In the results, please indicate analyses to detect the AAV construct (as opposed to mRNA or protein) in non-liver tissues.

Intraperitoneal injection was unsuccessful. Any speculation as to why?

The pictures in Fig. 3 are a bit dark, rendering the differences between wt and test a little hard to see. Do you have better pictures? If not, perhaps an inset at greater magnification would help make the point.

In lanes 3 and 4 of Fig. 1A and lane 5 of Fig. 1C, there is a band at the top of the western. Is this an artefact of each lane? An isoform? Cross-reactivity? Seen in liver but not other tissues? I can appreciate the desire to crop a Western to show only the "relevant" band (antibodies are not perfect - we all know that) but intellectual honesty requires the "whole" truth.

In Fig. 1D, it would help to provide a -/- (no AAV) value for SDO activity, to highlight the improvement with AAV. I gather that this could not be done in Fig. 1G, but how about Fig. 2D?

Finally, if direct damage to endothelial cells is a major feature of EE, why not show a comparison of this tissue (e.g. in blood vessels) in wt, -/-, and AAV mice?

1st Revision - Authors' Response 08 June 2012

Referee #1:

The authors present a convincing case that a mouse model of ethylmalonic aciduria can be treated with a recombinant human viral vector.

Figure 1D. We need to see SDO levels in untreated mice, to be convinced that the mechanism is due to the restoration of SDO activity.

Ibid in Fig 2D

Thank you we agree. SDO activity in untreated Ethe1-/- mice is now included in the figure as suggested.

How do the authors explain the multi-tissue rescue implied by Figure 3, given the highly tissue

specific expression of the human protein shown in Figure 1A? Presumably ALL of the non-hepatic effects are due to the removal of H2S, which creates the impression of improved OXPHOS? To be convincing, we really need to see formal measurement of the respiratory chain activity in these tissues, and parallel measures of H2S in these tissues. At present we are left with uncertainty on this issue.

Yes the Reviewer got our point. Based on the pathogenic mechanism proposed for the disease, we hypothesize that inhibition of COX, which by the way is only one of the pathogenic mechanisms acting in EE, is inhibited by H2S and can be recovered by promoting the clearance of this compound in circulating blood by conferring biochemical competence to a key-filtering organ, the liver. We now confirmed the (partial but significant) recovery of COX not only by looking at the histochemical COX reaction in tissues (Figure 3) but also by quantitative analysis of several individuals (Fig. 2F and Supplemental Table I). COX activity, which was measured in brain and skeletal muscle of 5 treated mice, doubled as compared to untreated KO mice (n. 3). The direct measurement of H2S is very cumbersome, aleatory and erratic, essentially because this compound is an unstable, extremely reactive reducing agent, volatile and therefore difficult to trap, and many methods do not distinguish the free form from thiols associated with proteins and other substrates. For any practical purposes it is in fact unfeasible, but easily and faithfully replaced by the measurement of thiosulfate, a stable biomarker of H2S. Thiosulfate accumulates in plasma of Ethe1 ko animals, were it can be easily measured by HPLC. Thiosulfate was markedly reduced in treated KO mice vs. untreated ones, almost to wt levels in the longer surviving AAV-treated group (Figure 2). However, thiosulfate levels in tissues are much lower than in plasma, and close to the detection limit of our HPLC-based method; this is true particularly for the brain, a limitation that prevented us from carrying out the assay in this tissue; nevertheless, we were able to measure thiosulfate in skeletal muscle (now shown in Supplemental Figure 4), with similar results as those obtained in plasma: whilst the levels were almost undetectable in wt mice, they were high in untreated KO mice, and significantly lowered in treated KO animals.

Have the authors tried allogenic bone marrow transplantation in their mice?

We are currently experimenting BMT in our mouse model. I twill take some time to collect convincing and robust data on this approach that we certainly are eager to obtain and share with the scientific community.

The title should not include "remarkably", which is a subjective term.

OK. Title amended.

Referee #2:

This paper by Di Meo et al describes a new strategy of treatment of ethylmalonic encephalopathy, a so far always fatal disease in humans, in a mouse model of the disease where the Ethe1 gene has been invalidated, by using AAV2/8 viruses to transduce the liver cells of the mice with the wild type human ETHE1 gene. Authors provide convincing evidence that the treatment of Ethe1-/- KO mice with AAV2/8-ETHE1 viruses allows Ethe1-/- mice to survive much longer than untreated mice, can restore their SDO enzymatic activity in liver to normal levels, as well as their blood level of thiosulfate, which in the disease directly correlates with the accumulation of toxic H2S due to deficiency in SDO activity.

The paper is clear and well written, and provides very interesting perspectives of clinical trials in patients by the use of similar AAV vectors. The referee feels that this manuscript will become suitable for publication in EMBO Molecular Medicine provided that authors answer the following

recommendations:

- *Authors used the strategy to target liver as the main filtering organ after blood circulation from intesine, one of the major productor of H2S. However, as said in the manuscript, H2S is also produced (at much lower amounts) to be used in many tissues, including CNS, as "gazotransmitter". Do authors know how, if yes, ETHE1 is expressed in the brain, and whether the invalidation of ETHE1 expression in the brain of patients may be responsible of the neurological symptoms independently of the liver defect? This concept is emerging now for numerous inborn errors of metabolism, and may be very important to consider for the long term outcome of patients. In particular, would excess production of H2S in CNS be evacuated to the bloodstream through blood-brain barrier, or would this excess H2S be trapped in the brain and be harmful here directly? In case of an endogenous ETHE1 expression in CNS, correction of the liver enzyme by AAV2/8 may appear insufficient to treat patients on the long term range. Authors may comment on this in their discussion.*

Ethe1 is ubiquitously expressed, as now stated in the introduction.

We previously produced a brain-specific Ethe1-/- mouse (Di Meo, 2011), which showed brain-specific COX deficiency, but not overt clinical phenotype. This raises the possibility that in humans, where COX deficiency seems to be more detrimental than in mice, local production of H2S can cause damage. H2S is a volatile compound, promptly crossing lipid bilayers and diffusing into the bloodstream. It is also freely permeant to the BBB. However, we want to stress the fact that the aim of this work is not to merely correct a liver defect, as this organ is not (clinically) affected by the disease in both EE patients and mice, but to rather use the liver as a detoxifying organ by re-establishing SDO competency and thus lowering the burden of circulating H2S. Accordingly, we observed both a reduction of the systemic biomarker thiosulfate in plasma (and skeletal muscle, see Supplemental Figure 4 and comments to Referee #1), and a recovery of COX activity in the tissues, including the brain.

- *How are the AAV2/8 viruses purified in terms of full viruses (including genome) and empty particles? Did authors analyze the potential effects of control (i.e. non-transducing ETHE1) AAV2/8 viruses in their mouse model? AAV2/8 per se may have toxic effects in various tissues, and this very important control seems to lack in the manuscript.*

Although we did not quantify the full/empty particles ratio in our research-grade vectors, this measurement was carried out previously during the standardization of the purification procedure in the laboratory of TIGEM (Alberto Auricchio); according to the optimized protocol, viruses are purified by two sequential rounds of Cesium Chloride ultracentrifugation that eliminate most of the empty particles from the final product.

We did not analyze the potential side effects of AAV2/8 in liver of Ethe 1 KO mice. However in previous studies we did not observe AAV2/8 side effects at doses up to 4x10e13 gc/kg in normal rodents (rats and mice) as well as in mice affected with lysosomal storage disease in liver (Tessitore et al, Mol Ther 2008; Cotugno et al Hum Gen Ther 2011; Cotugno et al PLOS ONe 2012) .

- *Kaplan-Meier survival curves in supplementary figures 1 and 2 should be better explained, in particular what represent the different colored curves.*

Supplemental figures 2 and 3 (previous Supplemental Figures 1 and 2) have been explained in more detail in the corresponding legends.

- Supplementary figure 1B: please add that this is (probably) a liver extract.

OK

Referee #3:

EE is an autosomal recessive fatal disorder due to mutations in ETHE1, which encodes sulphur dioxygenase, a mitochondrially-targeted protein involved in H2S detoxification. Mice lacking Ethe1 die within a few weeks. The authors report here of a dramatic increase in lifespan and in various aspects of Ethe1-related biochemistry and morphology in mice treated with a high titer of AAV expressing WT-Ethe1 under a liver-specific promoter.

This is a really nice paper demonstrating the potential and power of targeted gene therapy to treat, and perhaps even cure, an otherwise fatal monogenic disorder. The work has been done well and is convincing. I have only a few comments, mainly for the benefit of the reader.

Although reported elsewhere, it would be helpful to show, probably in Fig. 1, the pathway of H2S detoxification and where ETHE1/SDO fits into this scheme. Similarly, it would be informative to show the AAV construct in general outline (e.g. vector, promoter, gene, any relevant regulatory elements, location of pCR primers used to confirm DNA/mRNA).

The schemes of the metabolic pathway and AAV construct have been added as Supplemental Figure 1A and B, respectively.

Please note in the introduction in what tissues SDO is normally synthesised.

OK, SDO is an housekeeping genes, this is now made clear in the Introduction.

In the results, please indicate analyses to detect the AAV construct (as opposed to mRNA or protein) in non-liver tissues.

OK. We also performed PCR analysis on total DNA using the oligos (P1-P3) now specified in Supplemental Figure 1B.

Intraperitoneal injection was unsuccessful. Any speculation as to why?

We hypothesize that virions are impermeable to the peritoneal membrane.

The pictures in Fig. 3 are a bit dark, rendering the differences between wt and test a little hard to see. Do you have better pictures? If not, perhaps an inset at greater magnification would help make the point.

OK. Figures have been improved. We think that lower magnification gives a more general view of tissue morphology.

In lanes 3 and 4 of Fig. 1A and lane 5 of Fig. 1C, there is a band at the top of the western. Is this an artefact of each lane? An isoform? Cross-reactivity? Seen in liver but not other tissues? I can appreciate the desire to crop a Western to show only the "relevant" band (antibodies are not perfect - we all know that) but intellectual honesty requires the "whole" truth.

The band is an unspecific protein species present in all tissues. We are not aware of other isoforms of this protein. To be adamant, as rightly required, we changed the figure showing the whole blot. Note that the figure numbers are changed.

In Fig. 1D, it would help to provide a -/- (no AAV) value for SDO activity, to highlight the improvement with AAV. I gather that this could not be done in Fig. 1G, but how about Fig. 2D?

OK. Fixed. Note the figure numbers are changed.

Finally, if direct damage to endothelial cells is a major feature of EE, why not show a comparison of this tissue (e.g. in blood vessels) in wt, -/-, and AAV mice?

We recently reported that Ethe1-/- mice show a milder vessel damage than the brain (and other organs) in humans. This is possibly due to the intrinsic species specificity and very short lifespan of affected animals. Given the scarcity of anatomically overt vascular lesions in KO mice, hardly any improvement can be expected in treated animals (in fact our numbers are non significant and were omitted, but the methodological difficulty has been added in the Discussion).

2nd Editorial Decision 29 June 2012

Thank you for the submission of your revised manuscript "Effective AAV-mediated gene therapy in a mouse model of Ethylmalonic Encephalopathy" to EMBO Molecular Medicine. We have now received the enclosed reports from the referee that was asked to re-assess it.

As you will see, the reviewers acknowledge that the manuscript was significantly improved during revision. However, while reviewer #2 indicates that the manuscript is suitable for publication, reviewer #1 raises an issue that should be addressed. While we agree with the reviewer that COX activity in skeletal muscle seems to be unevenly distributed in the provided images, we acknowledge that the generation of new images (if not available) would be time-consuming. Should you be able to provide the data, we would encourage you to include them into the present study. Otherwise, we would strongly encourage you to include Table 1 into Fig 3 to address the raised concern.

On a more editorial note, please adjust the figure legend of Fig 3 so that it conforms to EMBO Molecular Medicine standards (please see any published EMBO Mol Med paper for examples).

I look forward to reading a new revised version of your manuscript as soon as possible.

Yours sincerely,

Editor
EMBO Molecular Medicine

***** Reviewer's comments *****

Referee #1:

The authors have addressed the majority of my concerns. However, I am not still convinced by the "partial reversal" of the defect as demonstrated histochemically in the final figure. The authors clear do not have better images. The ones presented have a curious artifact apparent on both cases and controls, showing a gradation in COX activity across the image, and an unusual fibre-type grouping/clustering effect across the image (ie the COX negative figres are not evenly distributed, as one would expect them to be). The authors should either provide better images, or remove this data

(not just the images, but the comment on the histochemistry in the results and discussion)

Referee #2:

This paper by Di Meo et al has now been correctly, and convincingly, revised and modified accordin to the demands of referees, and appears now acceptable for publication in EMBO Mol Med.

2nd Revision - Authors' Response 02 July 2012

Referee #1:

The authors have addressed the majority of my concerns. However, I am not still convinced by the "partial reversal" of the defect as demonstrated histochemically in the final figure. The authors clear do not have better images. The ones presented have a curious artifact apparent on both cases and controls, showing a gradation in COX activity across the image, and an unusual fibre-type grouping/clustering effect across the image (ie the COX negative figres are not evenly distributed, as one would expect them to be). The authors should either provide better images, or remove this data (not just the images, but the comment on the histochemistry in the results and discussion)

Answer:

We thank Referee #1 for the comment: we clearly have been collecting histochemical data from several different sections and animals; in the new figure 3 some panels for skeletal muscle specimens have been replaced so as to improve the quality and clarity of the results. We are sure that both Referee #1 and the Editor will agree with us that there is an obvious difference in the COX and SDH histochemical reactions among control, untreated and treated KO samples. As for the fiber-type grouping/clustering noticed by Referee#1, this array is, in our experience, a common finding in skeletal muscle of the mouse, which does not imply a reshaping of the motor unit, due, for instance, to denervation/reinnervation processes.

© EMBO
