# Supplementary material for: Effective AAV-mediated gene therapy in a mouse model of ethylmalonic encephalopathy
Source: EMBO Mol Med. 2012 Aug 20;4(9):1008–14. doi: 10.1002/emmm.201201433 (PMC3491831; doi:10.1002/emmm.201201433)
Supplement: Supplementary file 2 [file emmm0004-1008-SD2.pdf]

Supplemental Material Online

Contents:

Supplemental Figure 1: Explanatory schemes. (A) proposed mitochondrial pathway of H_2S disposal; (B) AAV2/8-TBG-*h.Ethe1*^{HA} construct.

Supplemental Figure 2: Characterization of *Ethe1*$^{-/-}$ mice treated with $4x10^{12}$ vg/Kg AAV2/8-TBG-*h.Ethe1*^{HA}.

Supplemental Figure 3: Kaplan-Meier survival probability curves.

Supplemental Figure 4: Thiosulfate concentration in skeletal muscle.

Supplemental Table I: COX/CS activities in skeletal muscle and brain.

Supplemental Figure 1

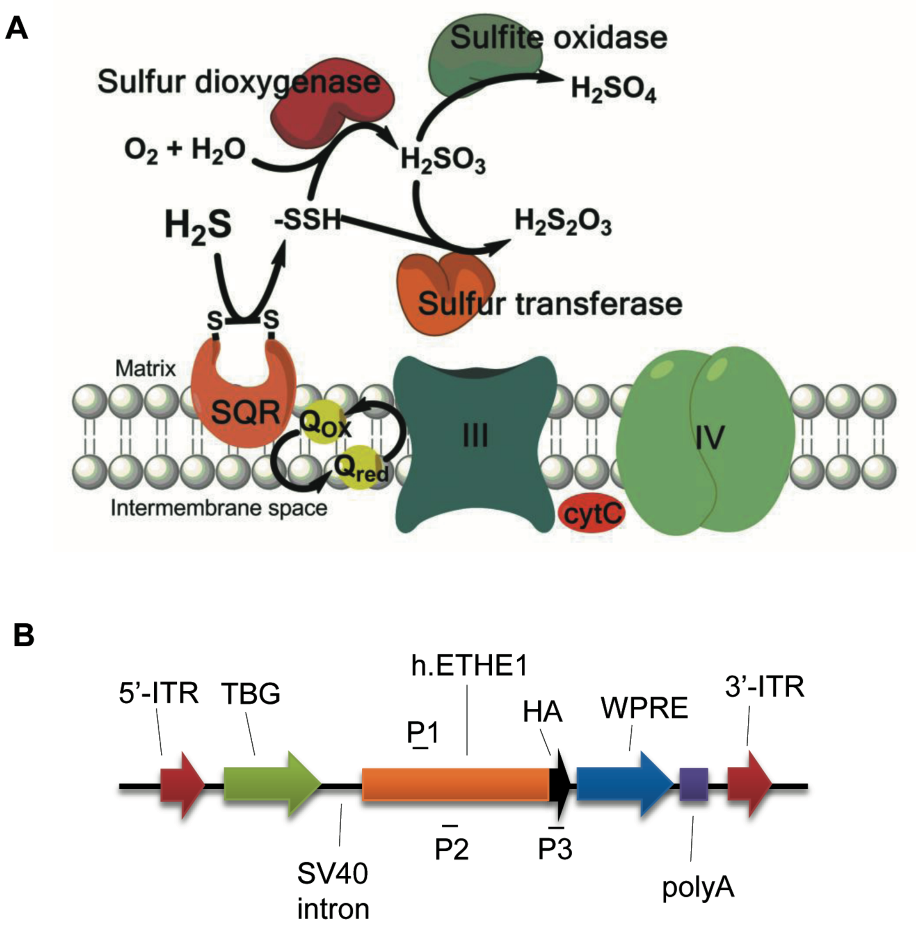

Supplemental Figure 1. Explanatory schemes. (A) Proposed mitochondrial pathway of H_2S disposal according to Hildebrandt and Grieshaber, 2008. SQR: sulfide:quinone oxidoreductase; Qox: oxidized coenzyme Q; Qred: reduced coenzyme Q; III: complex III; IV: complex IV; cytC: cytochrome C; the Sulfur dioxygenase is encoded by *Ethe1*. (**B**) AAV2/8-TBG-*h.Ethe1*^{HA} construct. ITR: inverted terminal repeat; TBG: tyroxine-binding globulin promoter; WPRE: Woodchuck Hepatitis Virus (WHP) Posttranscriptional Regulatory Element; P1-P3: primers used for PCR amplification.

Supplemental Figure 2

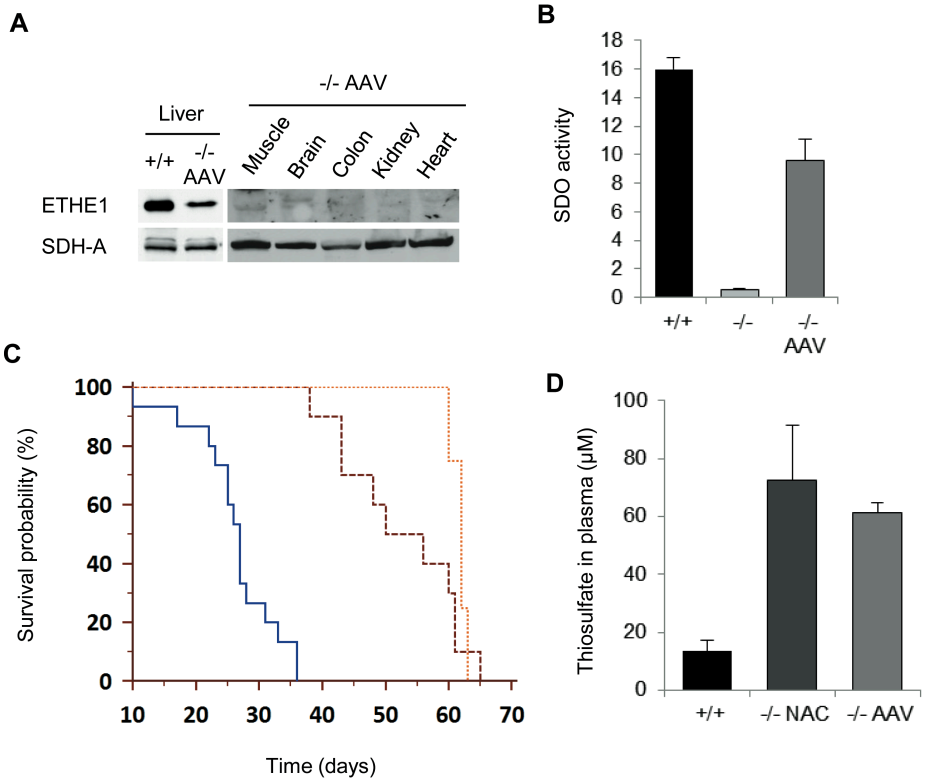

Supplemental Figure 2. Characterization of *Ethe1*$^{-/-}$ mice infected with 4x10^{12} vg/Kg AAV2/8-TBG-*h.Ethe1*^{HA}. (**A**) Liver-specific expression of the *h.Ethe1*^{HA} gene product using an anti-Ethe1 antibody; SDH-A is used as a protein load standard. (**B**) SDO activity, expressed as nmol O_2/min/mg) in *Ethe1*$^{+/+}$ *(+/+), Ethe1*$^{-/-}$ *(-/-),* and AAV-treated *Ethe1*$^{-/-}$ (-/- AAV) liver homogenates. *(***C***)* Kaplan-Meier survival probability curve. Significance was assessed by the log-rank test. Blue line: *Ethe1*$^{-/-}$ mice; Red line*:* NAC-treated *Ethe1*$^{-/-}$ mice*;* Yellow line: AAV+NAC-treated *Ethe1*$^{-/-}$ mice; the curves of NAC-treated and AAV+NAC-treated *Ethe1*$^{-/-}$ mice do not differ significantly; the difference with the untreated group is p<0.0001. **D**) Plasma thiosulfate: the levels in AAV+NAC-treated *Ethe1*$^{-/-}$ (-/- AAV) mice do not significantly differ from those in NAC-only treated (-/- NAC) animals. The difference with the untreated group is p<0.0001 (unpaired, two-tail Student's *t* test). See main text for details.

Supplemental Figure 3

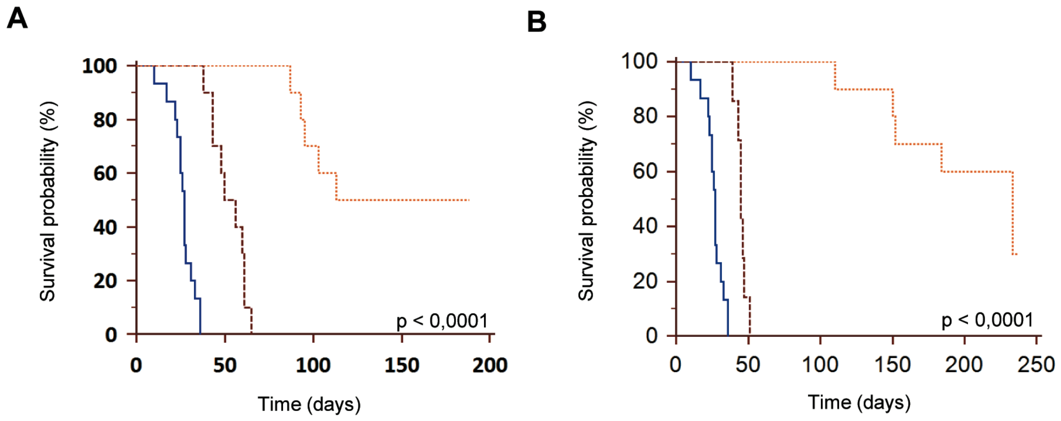

Supplemental Figure 3. Kaplan-Meier survival probability curves. (**A**) Blue line: untreated *Ethe1*$^{-/-}$ mice (n=15); Red line: NAC-treated *Ethe1*$^{-/-}$ mice (n=10); Yellow line: AAV+NAC-treated *Ethe1*$^{-/-}$ mice (n=10). NAC was administered throughout the experiment. (**B**) Blue line: untreated *Ethe1*$^{-/-}$ mice*;* Red line: NAC-treated *Ethe1*$^{-/-}$ mice (n=7); Yellow line: AAV+NAC-treated *Ethe1*$^{-/-}$ mice (n=10). NAC was suspended at P28. The two curves of NAC-treated *Ethe1*$^{-/-}$ mice are not significantly different.

Supplemental Figure 4

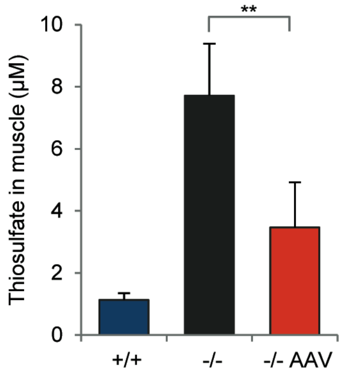

Supplemental Figure 4. Thiosulfate in skeletal muscle. Blue bar: *Ethe*$^{+/+}$ control mice (n=3); Black bar: untreated *Ethe1*$^{-/-}$ mice (n=3); Red bar: AAV-treated *Ethe1*$^{-/-}$ mice (n=5). ** Unpaired, two-tail Student's *t* test p= 0.0006.

Supplemental Table I

Tissue	Genotype	COX/CS*
Muscle	+/+	99.7 ± 5.0
	-/-	22.7 ± 2,1
	-/- AAV	46.4 ± 6.0
Brain	+/+	60.7 ± 5.5
	-/-	16.7 ± 2.5
	-/- AAV	32.8 ± 4.7

Values are expressed as means ± SD. +/+: *Ethe*$^{+/+}$ control mice (n=3); -/-: untreated *Ethe1*$^{-/-}$ mice (n=3); -/- AAV: AAV-treated *Ethe1*$^{-/-}$ mice (n=5)

*Student's *t* test between each group in each tissue p<0.001
